# Industry 4.0 Engineering Product Life Cycle Management Based on Multigranularity Access Control Model

**DOI:** 10.1155/2022/3655621

**Published:** 2022-01-21

**Authors:** Longfei Yu, Shifan Zhu

**Affiliations:** College of Mechanical and Electrical Engineering, Harbin Engineering University, Harbin 150001, China

## Abstract

In order to improve the management efficiency of the safety status of Industry 4.0 engineering products, the multigranularity access control model (MGACM) Industry 4.0 engineering product life cycle management (PLM) is adopted to optimize the safety management mode of Industry 4.0 engineering products in this paper. The multigranularity access control model is constructed in this paper, which has strong nonlinearity and better fault tolerance. In addition, the parameters of PLM are optimized through the multiparticle access control model, and PLM search is enabled. Taking into account the slow and easy convergence of the multigranular access control model, a niche technology with full life cycle heterogeneity and elimination mechanism is proposed to solve the premature convergence problem of the multigranular access control model. The final simulation results of this paper show that, compared with traditional algorithms, the proposed multigranularity access control model is more reliable and effective and has faster convergence speed and higher management efficiency.

## 1. Introduction

With the continuous advancement of society and unceasing enhancement of people's needs, data information has gradually become the basic technology for people's daily life, and the role of Industry 4.0 engineering product life cycle in people's lives has become more prominent. There are Industry 4.0 engineering products [1, 2], wireless communication Industry 4.0 engineering products, etc. Therefore, the Internet is usually used to transmit and receive data and information, and the quality of Industry 4.0 engineering products is more important. According to research at home and abroad, it is shown that the quality problems of industrial products are not simply solved by the use of preventive methods [3]. Under this circumstance, the security status perception of the integrated technology Industry 4.0 engineering products that need to obtain and process security information has attracted attention. However, Industry 4.0 engineering product safety situation management is an emerging technology, and many research contents have not been resolved so far. In order to provide more effective and accurate management, an improved multigranularity access control model is introduced to optimize the life cycle management of Industry 4.0 engineering products in this paper. First of all, PLM with better nonlinear ability and approximation speed is adopted instead of BP or RBF neural Industry 4.0 engineering products for management. Secondly, the multigranularity access control model is used to optimize the parameters of the neural Industry 4.0 engineering product, which has strong global search capabilities.

By constructing a multigranularity access control model, the access host granularity and access level granularity are optimized and processed. Meanwhile, the life cycle granularity and authorization distribution control granularity of industrial products are used, to complete the optimization process of the model and complete the realization of multigranularity access control. Finally, the analysis of the experimental results shows that the constructed model can effectively reduce the fault tolerance rate of the industrial product life cycle management system. Meanwhile, it can also strengthen the accuracy and completeness of model access control, which can better meet the special needs of PLM for access control.

## 2. Granularity Analysis of Access Control for Industry 4.0 Engineering Product Life Cycle Management

### 2.1. Granularity of Access Subject Composition

**Figure 1:**
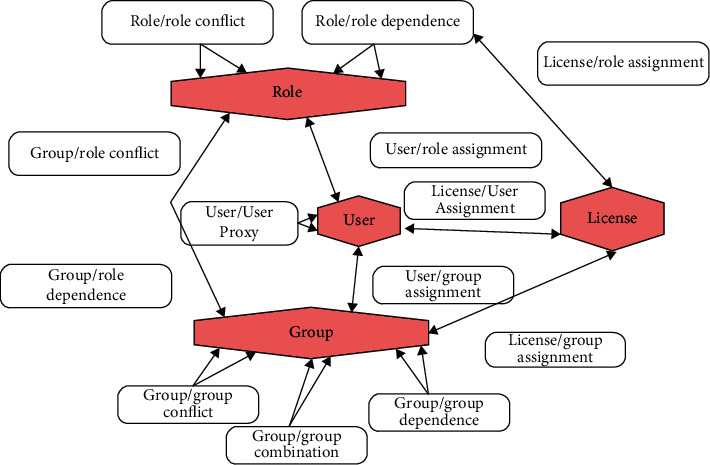
Access subject of multigranularity access control model MAC makes up the granular model.

The subject that can be accessed by the multigranularity access control model only allows two dimensions: role and user. This article implements its subject by increasing the granularity diameter. Granular composition, as a collection of multiple users, can achieve the completion of its architecture through a combination of different forms. The full range of permissions for function groups and users is open. As the authorizer of the granularity of the access subject, ASD can assign its authority to the restricted relationship with the same structure. If a user is assigned to group A, then the role permissions of group B can be obtained. For example, technicians in the assembly process group can assign permissions to them in accordance with the engineering product management standards of Industry 4.0, and the same user can be assigned different users/assigned roles.  Figure 1 shows the granularity of the access control subject and its relationship model.

The authorization of the access subject group can simplify the public permission distribution method for the unused functions in the same unit and can simplify the access control of the temporary project group. Because the user authorization can effectively improve the problems of personnel management, it is effective to reduce the confusion of the role. ASC and ASD are in a static relationship. At the same time, before assigning users to corresponding roles, their roles can be set according to the business rules of industrial products or possible problems can be solved in advance. ASC can make corrections when there is an error in the user's role assignment. The main function of ASD is to simplify the problem of repeated assignment of permissions for different roles. Therefore, the granularity of the access subject and its corresponding relationship can not only simplify its authorization procedure, but also avoid the probability of false authorization [4].

### 2.2. Access Object Level Granularity

**Figure 2:**
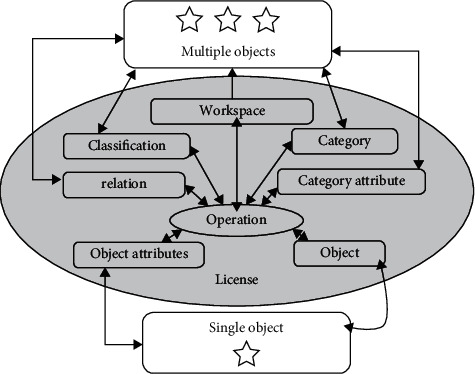
The process for multigranularity access control model MAC access to different object levels.

The life cycle management analysis system uses the engineering product sequence information to analyze the life cycle management parameters of the camera based on a specific model. Most of the calculated amount of the engineering product stability algorithm is here, and the accuracy of life cycle management parameters affects the accuracy of the stable engineering product, so it is an important part of the engineering product stability system [5]. The multigranularity access control model MAC is shown in Figure 2 on the visitor layer. Operations (OpeRation, OPAR) can be used in different types of granularity groups. For any optimal granularity, the achievable results can be used in special scenarios. During Industry 4.0 engineering product life cycle management process, due to the camera shaking during use, the engineering product sequence obtained by shooting will be jittered. The engineering product processing interference caused by this jitter will promote the engineering product life cycle management analysis technology emerging as the times require. Engineering product life cycle management analysis technology has been widely used in many fields of military and civilian use. From the perspective of military applications, large-scale guidance, fire control systems, and small self-seeking missile leading engineering products all involve engineering product life cycle management analysis technology. This technology can be used to improve the attack performance of weapon systems and for improvement of relevant indicators of other operations. In the photography of aviation field and terrain surveying and mapping instruments, in order to ensure that the corresponding measurement benchmarks for life cycle management analysis are obtained in the image plane of the instrument, the accuracy and processing speed of the measurement results are improved. As a new engineering product life cycle management analysis model that has gradually emerged in recent years, the multigranularity access control model can be used for the processing and analysis of Industry 4.0 engineering product life cycle management. The basic principle of this model is to use a different model for any Industry 4.0 engineering product data or perform multiple clustering operations on engineering products under different conditions and select the appropriate method for the calculation results obtained by the clustering operation, clustering and optimizing the data to obtain the best results.

### 2.3. License Allocation Control Granularity

As a bridge between the operation/object and the accessible subject, the license can realize comprehensive and neat distribution control. The particle progress control can ensure the consistency of the target to be visited. The distribution control particle size of the license can be distinguished according to the four aspects of the controllable control direction within the allowed range, the allowed access restriction, and the license priority. An Industry 4.0 engineering product life cycle management method is proposed. While transforming the two-dimensional engineering product life cycle management to one-dimensional engineering product data, it also evaluates and analyzes the deviation of the engineering product sequence in the life cycle management data, to eliminate the impact of engineering product quality due to lower sequence.

Allowable range, control direction, and access restrictions are static attributes, and allocation permissions need to be set (Table 1). Priority is a dynamic attribute, and the value before system execution is empty. If the system runs and loads all the permissions of the current user, the permissions are sorted according to the allowed source code and the priority of the above permissions. For example, the priority of direct permission from all users is 0, and the priority of permission from the user's group is 1.

## 3. Multigranular Access Control Analysis

### 3.1. Control Subject and Object and Related Analysis

First, the gradient descent training of error backpropagation is used to select and extract cluster members for the life cycle management of Industry 4.0 engineering products to provide an accurate data basis for subsequent cluster fusion. There is the actual sequence {*x*(*t*_0_+*i*Δ*t*)} of the information flow of the life cycle management Industry 4.0 engineering product data and *i*=0,1,…, *N* − 1. After setting *X* and *Y* as the Industry 4.0 engineering product life cycle management attribute set, the cluster space state vector calculation formula of the Industry 4.0 engineering product life cycle management is

(1) $$\begin{array}{c} X=\left[x\left(t_{0}\right),x\left(t_{0}+\Delta t\right),\ldots ,x\left(t_{0}+\left(K-1\right)\Delta t\right)\right]=\\ \left[\begin{array}{cccc} x\left(t_{0}\right) & x\left(t_{0}+\Delta t\right) & \cdots & x\left(t_{0}+\left(K-1\right)\Delta t\right)\\ x\left(t_{0}+J\Delta t\right) & x\left(t_{0}+\left(J+1\right)\Delta t\right) & \cdots & x\left(t_{0}+\left(K-1\right)J\Delta t\right)\\ \vdots & \vdots & \vdots & \vdots \\ x\left(t_{0}+\left(m-1\right)J\Delta t\right) & x\left(t_{0}+\left(1+\left(m-1\right)J\Delta t\right)\right) & \cdots & x\left(t_{0}+\left(N-1\right)\Delta t\right) \end{array}\right]. \end{array}$$

In the formula, *x* (*t*) represents the time series of the life cycle management information flow of Industry 4.0 engineering products. *J* represents the phase space time window function of the life cycle management reconstruction of the Industry 4.0 engineering product data and *m* represents the life cycle management cluster adjustment factor of the target engineering product data. Δ*t* represents the sampling time interval of Industry 4.0 engineering product data.

Then the calculation expression of the discrete sample spectrum feature quantity for the life cycle management of Industry 4.0 engineering products is as follows:

(2) $$\begin{array}{c} X_{p}\left(u\right)=s_{c}\left(t\right)e^{j2\pi f_{0}t}=\frac{1}{T}\text{rect}{\left(\frac{t}{T}\right)}^{j2\pi \left(f_{0}t+Kt^{2}\right)/2}. \end{array}$$

The formula *s*_*c*_(*t*) represents the characteristic scalar time series of the life cycle management of Industry 4.0 engineering products. *e*^*j*2*πf*_0_*t*^ represents the discrete sample center of the Industry 4.0 engineering product life cycle management cluster.

(3) $$\begin{array}{c} u_{\left(i,d\right)}^{\left(k+1\right)}=\left\{\begin{array}{c} x_{\left(i,d\right)}^{\left(t+1\right)}f_{\text{titness}}^{t}<f_{\text{fitness}}^{*},\\ z_{\left(i,d\right)}^{\left(k+1\right)}f_{\text{titness}}^{t}\geq f_{\text{fitness}}^{*}, \end{array}\right) \end{array}$$

*f*_fitness_^*t*^ is the expression representing the reliability coefficient of the life cycle management of Industry 4.0 engineering products. *f*_fitness_^*∗*^ represents the reliability interval coefficient for the life cycle management of Industry 4.0 engineering products.

According to the calculation of formulas (2) and (3), the best solution vector matrix formula for the life cycle management of Industry 4.0 engineering products centered on data clusters is

(4) $$\begin{array}{c} \Sigma_{r}=\text{diag}\left(\sigma_{1},\sigma_{2},\ldots ,\sigma_{r}\right)\in R^{r\times r}. \end{array}$$

The formula *σ*_*r*_ represents the position of Industry 4.0 engineering product life cycle management to *k* + 1. *R*^*r*×*r*^ represents the actual matrix of Industry 4.0 engineering product life cycle management.

The diagonal vector indicator of the life cycle management of Industry 4.0 engineering products is close to the target solution, and the following conditions can be met:

(5) $$\begin{array}{c} \sigma_{1}\geq \sigma_{2}\geq \cdots \geq \sigma_{r}>0. \end{array}$$

**Figure 3:**
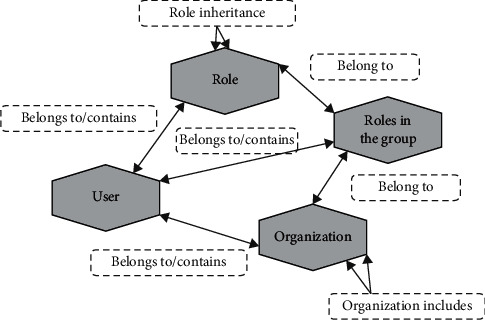
Correlative spread of common subjects.

**Figure 4:**
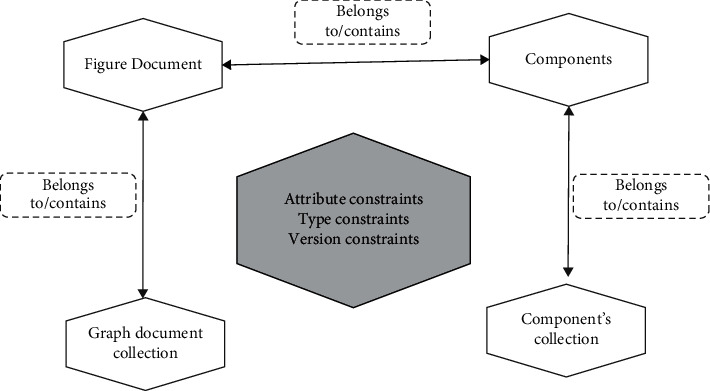
Correlative spread of common objects.

According to the granularity fusion, the IEPLCM system can be divided into related extensions of the control main control, as shown in Figures 3 and 4.

### 3.2. Analysis of Relevant Elements of Multigranular Access Control

Industry 4.0 engineering product life cycle management selection results, diversity and accuracy are used to define, comprehensive evaluate, and complete the selectivity of Industry 4.0 engineering product life cycle management. By searching the particle swarm in the Industry 4.0 engineering product life cycle management cluster to form the Industry 4.0 engineering product life cycle, the corresponding Industry 4.0 engineering product life cycle management data information feature vector *χ*_*i*_ is expressed as

(6) $$\begin{array}{c} l_{\varepsilon }\left(g\right)=\left(1-\rho \right)l_{\varepsilon }\left(g-1\right)+\gamma f\left(\chi_{i}\left(g\right)\right). \end{array}$$

In the above expression, *f* represents the corresponding adaptive function of the feature data feature vector *χ*_*i*_ of the life cycle management of Industry 4.0 engineering products. *γχ*_*i*_(*g*) represents the *ε*-th life cycle management corresponding media engineering product data optimization in the actual application process.

The expression of clustering *π*_*p*_ in Industry 4.0 engineering product life cycle management II is

(7) $$\begin{array}{c} \text{Acu}\left(\pi_{p}\right)=\text{NMI}\left(\pi_{p},\pi^{*}\right). \end{array}$$

In the formula, *π*_*p*_ and *π*_*q*_ represent the clustering integration of the life cycle management of Industry 4.0 engineering products. If there is less information shared with the Industry 4.0 engineering product life cycle management basic cluster, the accuracy of the basic cluster is low, and vice versa.

Based on the accuracy and diversity characteristics of the clusters based on the life cycle management of Industry 4.0 engineering products, the comprehensive evaluation criteria that define the clusters based on the life cycle management of Industry 4.0 engineering products include

(8) $$\begin{array}{c} \text{Eval}\left(\pi_{p}\right)=\lambda \text{Acu}\left(\pi_{p}\right)+\left(1-\lambda \right)\text{Div}\left(\pi_{p}\right). \end{array}$$

In the formula *λ* ∈ [0,1], the accuracy and diversity of the life cycle management of Industry 4.0 engineering products are an important degree in the comprehensive evaluation standard.

Based on the diversity Div(*π*_*p*_) of the basic clustering of Industry 4.0 engineering product life cycle management, formula (8) calculates the probability pro(*π*_*p*_) of selecting the basic clustering algorithm of each Industry 4.0 engineering product life cycle management as the optimized basic clustering. The calculation formula is as follows:

(9) $$\begin{array}{c} \text{pro}\left(\pi_{p}\right)=\frac{\text{Div}\left(\pi_{p}\right)}{\sum_{p=1}^{B}\text{Div}\left(\pi_{p}\right)}. \end{array}$$

The pro(*π*_*p*_) calculation result of the benchmark formula (9) is used to use roulette to randomly select the cluster based on the life cycle management of the Industry 4.0 engineering product and obtain the integration cluster of the Industry 4.0 engineering product life cycle management.

If there are *N* data in the life cycle management of Industry 4.0 engineering products, the attribute dimension of the data can be represented by *w*, and the position and velocity matrix of Industry 4.0 engineering product data can be represented by *N*:

(10) $$\begin{array}{c} \delta_{1}^{1}\delta_{1}^{2}\cdots \delta_{1}^{w}\delta_{1}^{1}\delta_{1}^{2}\cdots \delta_{2}^{w}\cdots \delta_{\tau }^{1}\delta_{\tau }^{2}\cdots \delta_{\tau }^{w}\delta_{1}^{1}\cdots \delta_{\tau }^{w}f. \end{array}$$

Industry 4.0 engineering product life cycle management particle group overall fitness decentralized

(11) $$\begin{array}{c} \psi^{2}=-\sum_{\eta =1}^{v}\left(\frac{f_{i}-f_{\text{avg}}}{f}\right). \end{array}$$

In the formula, *v* represents the number of particles of Industry 4.0 engineering product life cycle management. *f*_*i*_ represents the matching degree value of the *η*th Industry 4.0 engineering product life cycle management particle. *f*_avg*A*_ represents the average fitness value of current particles in the life cycle management of Industry 4.0 engineering products. *ψ*^2^ < *φ* represents the determination threshold of the Industry 4.0 engineering product life cycle management *φ* and can perform optimization of updating the position and velocity of Industry 4.0 engineering product data particles according to the following expressions:

(12) $$\begin{array}{c} v_{t}=\omega v_{t-1}+\kappa_{1}\times {\text{rand}}_{1}\left(\right)\left(\text{pbest}-x_{t-1}\right)+\kappa_{2}\times {\text{rand}}_{2}\left(\right)\left(\text{gbest}-x_{t-1}\right), \end{array}$$

(13) $$\begin{array}{c} x_{t}=x_{t-1}+v_{t}. \end{array}$$

### 3.3. Key Algorithms for Multigranular Access Control

As mentioned above, to judge authority through access objects and related analysis, we must fully consider the spreading mechanism between access objects. This solves the set of equivalent access subjects associated with the detection target user and the set of equivalent control clients corresponding to the control target instance. It also provides a basis for authentication based on access control permissions. Because the solver process that controls the set of equivalent access subjects and the set of objects is similar, the following only introduces the solver process of the set of equivalent access subjects. The pseudo-algorithm for solving the set of equivalent access subjects is described below.

The purpose of IEPLCM's multigranular access control is to temporarily allow or deny the request based on predefined permissions when a specific user requests access to specific product components or text data. When the access is determined to be a function, the entry parameter of the function is user *u*. In addition, the operation op performed by the requested access control client object functions as a function that returns *b* according to a predefined access rule. If the return value of the function is true, it can be executed [6, 7]. On the contrary, the access request is denied, and the corresponding access control is shown in Algorithm 2 based on the analysis of the abovementioned access control model components and masters, guests, and their related relationships pseudo-algorithm of the project process.

It can be seen from Algorithm 2 that the solver process for verifying access control authority is a process of multiple nested loops to ensure the efficiency of the algorithm. The number of permitted rule items sets common access control items for control guests (such as certain types of text, components) that have the meaning of data sets and sets special and individual access control items for specific control guests to achieve access control of data.

## 4. Product Life Cycle Management Process under the Multigranularity Access Control Model

### 4.1. Multigranularity Access Control Model

**Figure 5:**
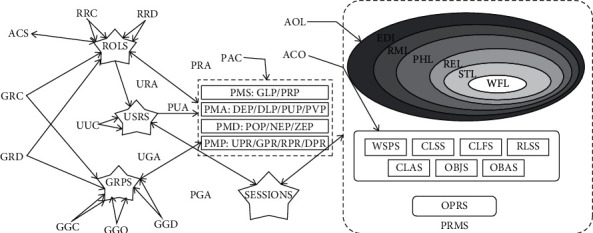
Multigranularity access control model and its elements.

It describes the composition granularity of the access subject, the level granularity of the access object, the granularity of the life cycle, and the license allocation control granularity from different aspects and levels to control the access to PLM. Based on the above analysis, as shown in Figure 5, a PLM access control model based on RBAC is constructed.

### 4.2. Formal Description of Multigranularity Access Control Model

**Figure 6:**
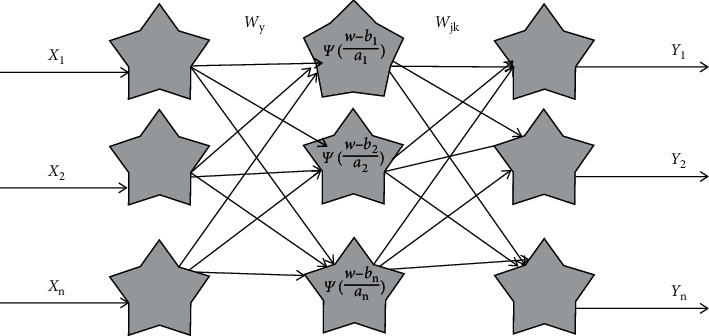
MGACM's Industry 4.0 engineering product topology.

MGACM usually adopts a closed type with three layers of feedforward neural Industry 4.0 engineering products. In the model in this paper, it is assumed that the Industry 4.0 engineering product topology is shown in Figure 6, and there are *m* nodes in the input layer, *h* nodes in the hidden layer, and *n* nodes in the output layer. The input samples are represented by *X*_1_ ~ *X*_*m*_ and the output samples are represented by *Y*_1_ ~ *Y*_*n*_. The stretching and translation parameters are expressed by *a*_1_ ~ *a*_*h*_*b*_1_ ~ *b*_*h*_, respectively. The link weights in Industry 4.0 engineering products between the input layer to the hidden layer and the hidden layer to the output layer are represented by *w*_11_ ~ *w*_*mh*_*w*_11_′ ~ *w*_*hn*_′, respectively.

For the hidden layer, Morlet is selected as the main wavelet function and its equation is as follows:

(14) $$\begin{array}{c} \psi \left(x\right)=\cos\left(1.75x\right)\exp\left(-\frac{x^{2}}{2}\right). \end{array}$$

The number *h* of hidden layer nodes is determined by the number *m* of input layer nodes. Calculation formula is

(15) $$\begin{array}{c} h=2m+1. \end{array}$$

It follows that

(16) $$\begin{array}{c} g\left(x\right)=\frac{1}{\left[1+\exp\left(-x\right)\right]}. \end{array}$$

The *x* in (1) and (3) represents the data of the previous layer.

The output result of MGACM based on wavelet function is

(17) $$\begin{array}{c} Y_{t}=\sum_{s=1}^{h}w_{st}^{\prime }\psi \left(\frac{\sum_{r=1}^{m}w_{rs}-b_{s}}{a_{s}}\right). \end{array}$$

Generally, the gradient descent method is used to calculate the connection weight of Industry 4.0 engineering products, and the parameters in MGACM are more accurate than before. The extreme points of this method are only approximate values. Meanwhile, it is easily troubled by the problem of the best local Industry 4.0 engineering products. Therefore, this article intends to use an improved multigranularity access control model to optimize the parameters of local Industry 4.0 engineering products.

The increase of the fitness value determines the development direction of the multigranular access control model. The error *E* of MGACM is defined as an individual fitness function:

(18) $$\begin{array}{c} E=\frac{1}{2}\sum_{t=1}^{n}{\left(Y_{t}-Y_{t}^{\prime }\right)}^{2}, \end{array}$$

(19) $$\begin{array}{c} f=\frac{1}{1+E}, \end{array}$$

where *Y*_*t*_ represents the real value of the *t*-th output node, and *Y*_*t*_′ represents the predicted output.

The most common selection operation is to choose roulette. However, the predictive model is selected with the expected value so as not to harm the best individual at the smaller number of samples [8, 9]. Expected value is calculated by (20). Therefore, the problem of probability is transformed into a problem of frequency. In the meantime, excellent personal retention institutions directly allow individuals to maintain the highest fitness value of the next generation of modern times.

(20) $$\begin{array}{c} q_{i}=\frac{f_{i}}{f_{\text{sum}}/N},\;i\leq N. \end{array}$$

This represents the sum of the overall individual fitness values. *f_sum_* represents the sum of all individual fitness values. *f_i_* represents the ith personal health value. *N* represents the total number of people. In addition, rounding is required by *q*_*i*_.

The choice of parameters is very important in the simulation process of the multigranularity access control model. For fixed values, the classic access control model always relies on crossover and mutation probabilities. However, it cannot dynamically adjust the probability of multigranularity in the evolution process, and the convergence speed is unstable. In addition, in order to deal with crossover operators and mutation operators, a multigranularity access control method is adopted in this paper. The multigranularity access control model probabilities *p*_*c*_ and *p*_*m*_ are defined in the following equations:

(21) $$\begin{array}{c} p_{c}=\left\{\begin{array}{cc} \alpha_{1}-\frac{\left(\alpha_{1}-\alpha_{2}\right)\left(f_{\max}-f^{\prime }\right)}{f_{\max}-f_{\text{avg}}}, & f^{\prime }\geq f_{\text{avg}},\\ \alpha_{2}, & \text{else,} \end{array}\right) \end{array}$$

(22) $$\begin{array}{c} p_{m}=\left\{\begin{array}{cc} \beta_{1}-\frac{\left(\beta_{1}-\beta_{2}\right)\left(f_{\max}-f\right)}{f_{\max}-f_{\text{avg}}}, & f\geq f_{\text{avg}},\\ \beta_{2}, & \text{else.} \end{array}\right) \end{array}$$

Among them, *α*_1_, *α*_2_, *β*_1_, *β*_2_, respectively, represent random values between [0, 1]. In this paper, *α*_1_=0.8, *α*_2_=0.5, *β*_1_=0.05, *β*_2_=0.001 are assumed. *f*_max_ represents the largest individual fitness value in the population. The average personal fitness value is represented by *f*_avg_. The parent fitness value before the crossover operation is expressed as *f*′. *f represents the fitness value suitable for the mutated individual.* Assuming that the initial multigranularity randomly generates *N* × *m* × *n* data, the complexity of the algorithm is *O*(*m*^2^ × *n*^2^).

Suppose there are *N* individuals with multiple granular dimensions. Depending on the size of the genetic factor, problems sometimes arise in real numbers. Therefore, the real number multigranularity should be normalized by the following function:

(23) $$\begin{array}{c} {\hat{x}}_{pj}=\frac{\left(x_{pj}-x_{pj\min}\right)}{x\left(x_{pj\max}-x_{pj\min}\right)}, \end{array}$$

where *x*_*pj*_ represents the multigranularity of the *p*-th individual at the *j*-th point in the genetic sequence.

After normalization by (23), the fuzzy similarity matrix *R* between individuals is created by

(24) $$\begin{array}{c} R_{pq}=\frac{\sum_{k=1}^{Chromlen}\min\left({\hat{x}}_{pk},{\hat{x}}_{qk}\right)}{\sum_{k=1}^{Chromlen}\max\left({\hat{x}}_{pk},{\hat{x}}_{qk}\right)} \end{array}$$

The fuzzy similarity matrix satisfies reflexivity and symmetry. According to [10, 11], the ambiguous equivalent matrix solves the problem of ecology degree more effectively. Therefore, by searching the fuzzy minimum transmission limit of the similar matrix *R*, the corresponding ambiguity equivalent matrix *T* is obtained in this paper, and the matrix is clustered as a whole.

If the similarity coefficient *λ* is less than the coefficient *T*_*pq*_ of each pair of individuals, that is, *λ* ≤ *T*_*pq*_, the individuals *x*_*p*_ and*x*_*q*_ are divided into the same niche, until all individuals are divided into appropriate positions.

(25) $$\begin{array}{c} \text{Niche}\left(k\right)⇐\left\{x_{p}\cdots x_{q}\right\}\left(1\leq k\leq \text{Chromlen}\right). \end{array}$$

Based on the fuzzy equivalent matrix and the total number, the similarity coefficients *λ* are dynamically updated as follows:

(26) $$\begin{array}{c} \lambda_{t}=\frac{\sum_{j=1}^{N}T_{\max j}}{N}. \end{array}$$

Among them, *T*_max*j*_ represents the equivalent coefficient between the individual with the maximum fitness value *x*_max_ and the individual *x*_*j*_.

In order to use the improved multigranularity access control model to quantitatively analyze the diversity, equation (27) is defined to calculate the multigranularity diversity.

(27) $$\begin{array}{c} d_{t}=-\sum_{n=1}^{Q}p_{n}\log\left(p_{n}\right), \end{array}$$

(28) $$\begin{array}{c} p_{n}=\frac{L_{mn}}{N}, \end{array}$$

where *Q* represents the number of submultigranularities in the *t*-th generation, *T*_max*j*_ represents the number of *n*-th submultigranularities, and *N* represents the total number of individuals of the species. *The higher the d, the greater the diversity.*

If the fitness value for a certain ecological location is much smaller than other values

(29) $$\begin{array}{c} \left|f_{i}-f_{\max}\right|<f_{\text{default}}, \end{array}$$

then

(30) $$\begin{array}{c} f_{i}=f_{\text{niche}}\left(i\right),\;1\leq i\leq n, \end{array}$$

(31) $$\begin{array}{c} f_{\text{niche}}=\left(f_{1}^{\prime },f_{2}^{\prime },f_{3}^{\prime },\ldots ,f_{n}^{\prime }\right), \end{array}$$

where *f*_max_ is the highest applicability value in the same generation. The default threshold of the adaptability value is *f*_default_. *f*_niche_(*i*) represents the fitness of the *i*-th individual.

### 4.3. The Permission Consistency Control Operation of the Multigranularity Access Control Model

The session of the multigranularity access control model refers to the process in which a specific user registers and applies the permissions he owns. Through different login methods, the session includes the current user global authentication registration, proxy user global authentication registration, current user project registration, and proxy user project authorization registration. If the login user is set to *u*, then for any registration method, user *u*'s permission distribution comes from the following 7 methods, allowing direct assignment to users.

(32) $$\begin{array}{c} PA_{u}\left(u\right)=u\_a\_ps\left(u\right). \end{array}$$

The licenses owned by the user's group:

(33) $$\begin{array}{c} PA_{ug}\left(u\right)=\underset{g\in u\_a\_gs\left(u\right)}{\cup }g\_a\_ps\left(g\right). \end{array}$$

The permissions owned by the user's role:

(34) $$\begin{array}{c} PA_{ur}\left(u\right)=\underset{r\in u\_a\_rs\left(u\right)}{\cup }r\_a\_ps\left(r\right),\\ PA_{gg}\left(u\right)=\underset{g\in \left(\underset{g_{y}\in u\_a\_gs\left(u\right)}{\cup }g\_d\_gs\left(g_{y}\right)\right)}{\cup }g\_a\_ps\left(g\right). \end{array}$$

The permission of the user's group on which the user relies:

(35) $$\begin{array}{c} PA_{gr}\left(u\right)=\left\{\left(p,PAC\right)|PAC.PMA=DE\;P\vee PAC.PMA=PUP\right\},\\ PA_{rr}\left(u\right)\subseteq \underset{r\in \left(\underset{r_{y}\in u\_a\_r\left(u\right)}{\cup }r\_d\_rs\left(r_{y}\right)\right)}{\cup }r\_a\_ps\left(r\right). \end{array}$$

The permission of the role that the user's group relies on, and

(36) $$\begin{array}{c} PA_{rr}\left(u\right)=\left\{\left(p,PAC\right)|PAC.PMA=DE\;P\vee PAC.PMA=PUP\right\},\\ PA_{rr}\left(u\right)\subseteq \underset{r\in \left(\underset{r_{y}\in u\_a\_r\left(u\right)}{\cup }r\_d\_rs\left(r_{y}\right)\right)}{\cup }r\_a\_ps\left(r\right). \end{array}$$

The permission of the group on which the user's role depends:

(37) $$\begin{array}{c} PA_{rr}\left(u\right)=\left\{\left(p,PAC\right)|PAC.PMA=DE\;P\vee PAC.PMA=PUP\right\}. \end{array}$$

Therefore, all the licenses of user *u* are allocated as

(38) $$\begin{array}{c} PA\left(u\right)=PA_{u}\left(u\right)\cup PA_{ug}\left(u\right)\cup PA_{ur}\left(u\right)\cup PA_{gg}\left(u\right)\cup PA_{gr}\left(u\right)\cup PA_{rg}\left(u\right)\cup PA_{rr}\left(u\right). \end{array}$$

**Figure 7:**
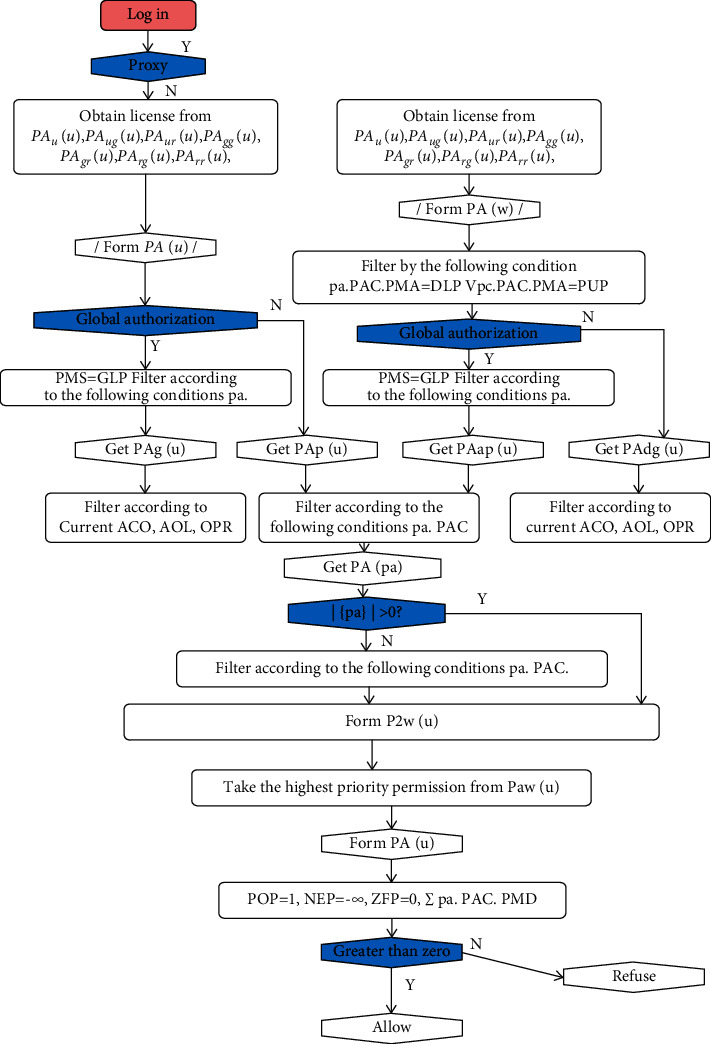
Multigranularity access control model MAC permission consistency control process.

License consistency control refers to the combined operation of all the licenses that have the same life cycle state, the same object, and the same operation, but the license distribution control combination operation of all permissions, to finally determine whether this operation is allowed to be executed [12, 13]. Let *PA* denote a single license distribution, and the license distributions in the four sessions are denoted as *PA*_*g*_(*u*), *PA*_*dg*_(*u*), *PA*_*p*_(*u*), and *PA*_*dp*_(*u*), respectively. The main flow of the permission consistency control algorithm is shown in Figure 7.

The main steps are described as follows:

1. *u* log in and load all the license distribution *P* (*u*) according to the seven aspects of license distribution. If the proxy is *u*′, all the licenses loaded into *u*′ are allocated to *P* (*u*′), and all the licenses that can be delegated to *P* (*u*′) need to be filtered out, that is, all the licenses owned by *u* as a proxy.
2. According to whether it is a global authorization, filter with the condition pa.PAC.PMS = GLP to form the current user global permission *PA*_*g*_(*u*), current user project permission *PA*_*p*_(*u*), proxy user global permission *PA*_*dg*_(*u*), and proxy user project permission *PA*_*dp*_(*u*).
3. If it is a global license, you only need to filter the license set according to the current ACO, AOL, and OPR and obtain all the license sets *P*_cur_(*u*) for the operation of the object in the current life state. If it is a project license, it must not only filter according to ACO, AOL, and OPR, but also filter according to pa.PAC.PMS = PRP, but if the obtained license set is null, the global license of the current object needs to be borrowed; that is, it will be filtered once based on ACO, AOL, and OPR filter again, to get *P*_cur_(*u*).
4. In view of *P*_cur_(*u*), according to the priority of license allocation, select all the license allocations *P*_pri_(*u*) with the highest priority. Then let the positive license be 1, the negative license is −] (can be replaced by a larger negative number), the zero license is 0, and the pa.PAC.PMD is solved in the sum *P*_pri_(*u*). If the result is greater than zero, the execution is allowed; otherwise the execution is refused [14, 15].

## 5. Examples and Results Analysis

### 5.1. Data Preprocessing

In order to verify the validity of the predictive model, the real security provided by the laboratory Industry 4.0 engineering product life cycle management platform is adopted in this paper. The data, based on the evaluation of the safety status of Industry 4.0 engineering products, effectively realized the prediction of the value of the safety status of Industry 4.0 engineering products.

The 90-day continuous data is selected from the product lifecycle management platform for experiment. This data can be divided into two parts. As a training sample 76 days ago, the remaining 14 days were used as a test sample. According to the analysis of security data, deep attacks will be carried out regularly within 5 days. Therefore, this paper uses 5 days as the vector dimension input and 1 day as the vector dimension output. The selection of the dimension of the security situation is shown in Table 2. Confirm that the prediction model of MGACM Industry 4.0 engineering product structure is 5-11-1.

The magnitude difference in the value of the safety situation of Industry 4.0 engineering products will affect the education of Industry 4.0 engineering products. In order to avoid this phenomenon, the safety status values of Industry 4.0 engineering products within 90 days are standardized as follows:

(39) $$\begin{array}{c} \hat{X}=\frac{\left(X-X_{\min}\right)}{\left(X_{\max}-X_{\min}\right)}. \end{array}$$

This includes the minimum value of the sample and the maximum safety status value of the Industry 4.0 engineering product. *X* is the safety status value of the standardized Industry 4.0 engineering products successively.

Select the absolute error (AE), and define the mean relative error (MR) and mean square error (RMMSE) as the criterion for predicting accuracy.

(40) $$\begin{array}{c} \text{AE}=\left|Y_{k}-Y_{k}^{\prime }\right|, \end{array}$$

(41) $$\begin{array}{c} \text{MRE}=\frac{1}{N}\sum_{k=1}^{N}\left|\frac{Y_{k}-Y_{k}^{\prime }}{Y_{k}}\right|, \end{array}$$

(42) $$\begin{array}{c} \text{RMSE}=\sqrt{\frac{1}{N}\sum_{k=1}^{N}{\left(Y_{k}-Y_{k}^{\prime }\right)}^{2}}, \end{array}$$

where *N* is the number of samples of the safety value of Industry 4.0 engineering products. It indicates the actual safety value of Industry 4.0 engineering products and displays the predicted value.

## 6. Results Analysis and Comparison

In order to prove the superiority of the proposed model, the MGACM, the performance of the multigranular access control model BP and that of the multigranular access control model MGACM are compared. Through the comprehensive training of these algorithms, we have selected convergence speed, diversity, and prediction accuracy to determine the advantages of these algorithms and verified the effectiveness of the proposed model.

**Figure 8:**
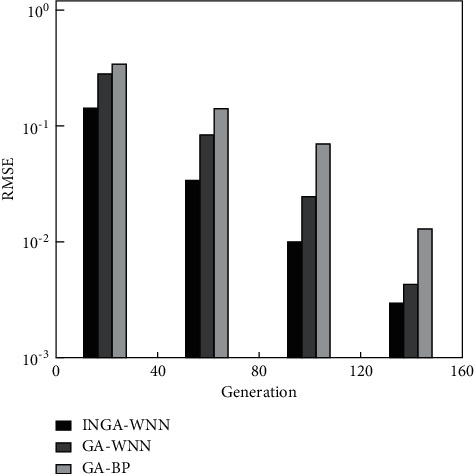
The convergence rate of the prediction model.

**Figure 9:**
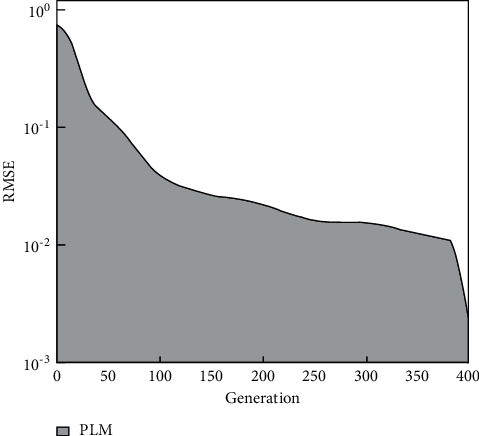
Convergence speed of PLM.

In Figures 8 and 9, the horizontal axis represents the generation, and the vertical axis represents the squared error. It can be seen from this paper that different algorithms have different convergence speeds. The multigranularity access control model has the fastest convergence speed, converging to the 68th generation. The second high-speed algorithm is the multigranularity access control model PLM, which ended in the 118th generation. Multigranularity access control models BP and PLM stopped operating in the 139th and 359th generations, respectively, achieving convergence accuracy. Therefore, in this paper, we conclude that the combination of the modified small environment technology and the PLM model effectively reduces the convergence time and improves the prediction efficiency.

Therefore, this article attempts to increase product diversity to improve the premature problem of the multigranularity access control model. Equation (42) is the quantitative analysis of product diversity. It can be seen from Figure 9 that the diversity value of MGACM is stable in the early stage around 0.69. Comparing the classic and access control model, MGACM has great advantages in maintaining product diversity.

## 7. Conclusions

This paper proposes an MGACM security situation management model for Industry 4.0 engineering products, which uses an improved multigranularity access control model to optimize parameters. This management model combines an improved security situation analysis method with a multigranular access control model. Therefore, the new security situation analysis method has improved optimization capability and convergence speed of the multigranular access control model. In addition, the improvement of product diversity effectively solves the problems of premature and low convergence. The experiment proves the reliability and validity of the multigranular access control model, which can also manage the security status of Industry 4.0 engineering products more accurately. Therefore, the multigranularity access control model MAC is suitable for large- and medium-sized enterprises, such as aviation and automobile enterprises, that have a wide variety of Industry 4.0 engineering products, complex structures, and strict access control.

## Figures and Tables

**Algorithm 1:**
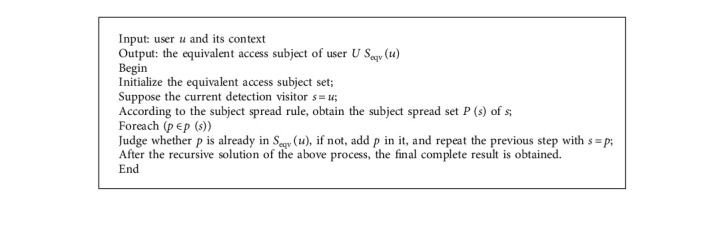
Equivalent access subject set solution.

**Algorithm 2:**
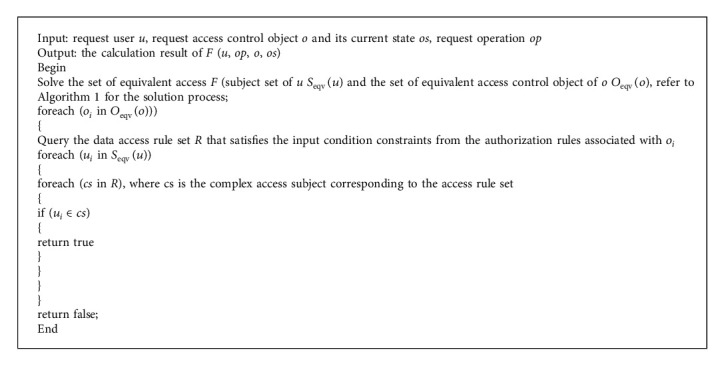
Access control authority verification solution.

**Table 1:** Access control direction combination relationship.

Control direction A	Control direction B	Combination result
Positive license	Negative license	Refusal
Positive license	Positive license	Allow
Positive license	Zero license	Allow
Negative license	Negative license	Refusal
Negative license	Zero license	Refusal
Zero license	Zero license	Refusal

**Table 2:** Selection of security status dimensions.

Input sample	Output sample
*X*_1_, *X*_2_, *X*_3_, *X*_4_, *X*_5_	*X*_6_
*X*_2_, *X*_3_, *X*_4_, *X*_5_, *X*_6_	*X*_7_
…	…
*X*_71_, *X*_72_, *X*_73_, *X*_74_, *X*_75_	*X*_76_

## Data Availability

The labeled dataset used to support the findings of this study is available from the corresponding author upon request.
